# Professional Standards

**Published:** 1904-01-15

**Authors:** N. S. Hoff


### PROFESSIONAL STANDARDS.

BY N. S. HOFF, D.D.S.

Read before the Central Michigan Dental Association, November 4, 1903.

Professional ideals or standards, so far as they apply to the professions which may be classed as ministerial in their functions, and all the professions of the healing art should certainly be so classified, should in application be characterized as beneficent or philanthropic. By this statement, we do not say that the physician or healer of whatever sort should freely give of his time and skill without compensation of any substantial kind; but, that the inability of the afflicted to make proper compensation should not necessarily prohibit the service. It sometimes seems that this principle is more often observed in its breach than in its realization. Too many professional men forget, as if they never knew, that there are nobler rewards than the mere dollars and cents to be had for so much per hour or completed service. They fail to appreciate that there can be a difference between the methods of the merchandizer and one who deals with the more sacred relations. They do not realize that one deals with the temporal and inconsequential affairs of life, while the other very often makes or mars the very issues of life. David Harum perverts the christian code of morals of "Do unto others as you would have them do unto you," by adding the significant words of a shrewd horse-trader," but do him first." That similar perversion of our professional code is general, I do not mean to say, but so many unprofessional acts come to one's notice, that it seems that some agitation of this subject in its relations to our work and calling may not be without beneficial influence. If not in actual correction, perhaps in stimulating more careful consideration of our relation to such as may entrust their very lives, it may be, to our faithful ministration.

Too many men choose their calling from wrong motives; they enter a sacred calling which calls for great sacrifices with no natural disposition to make concessions to any one except when forced to do so. They have either inherited, or acquired by faulty home environment, an instinct of selfishness which is wholly at variance with the lofty ideals which will make their lives noble and worthy in the practice of their profession. These faults of character are sometimes eliminated during the preparation for their work, if the students happen to fall into the hands of instructors who have proper conception of right relations, and appreciate the necessity for emphasizing their importance. Very often, however, a student will go through a whole college course and graduate without its ever occurring to him, that the one thing that is fundamental to the career upon which he is entering is the matter which he very often prides himself, he will strive to circumvent. He has perhaps been cheating himself all through his college course by believing he has done a real smart thing when he has succeeded in convincing his instructors that he was imbibing knowledge and accumulating skill, while all the time he has used his best efforts to follow Harum's code and do his teacher. The result of such a course is so obvious that we need not dwell further upon it, except to say that it sooner or later comes back upon the head as a boomerang, and accounts for much of the unprofessional conduct which we all meet continually and which seems at times to be on the increase. It is sometimes intimated that our educational institutions and examining boards are not doing their whole duty or these incompetent and irresponsible men would not be permitted to practice at all.

But strange as it may seem, while colleges and State Examining Boards are made up of clever people as a rule, they are not infallible judges of hidden character, and, sometimes, it may be they are culpably neglectful of their opportunities. These unfortunate traits of character or habits are very often developed after men begin practice, as a consequence of the hard competition and the peculiar environments into which they are forced by circumstances and which they seem unable to overcome. Many a man is driven to do cheap work or render inferior service because the people whom he serves have no proper conception of their obligations. And just here is where it seems that our profession must begin to do a work which is tedious but necessary, before the conditions which we now deplore can be changed. It is perhaps best, however, that we begin this reform within ourselves first, and then we may hope to influence more satisfactorily those who really are not to blame, because ignorant. We can, in my judgment, do nothing better as individuals and as a profession than to consider most carefully our professional ideals. I want to call your attention to a few of these ideals, which it seems to me are fundamental, but by no means inclusive, and which will serve to bring this subject more vividly before our minds for practical consideration. I would suggest that we may consider the bearing of the subject from its *Artistic, Professional* and *Commercial Relations.* What then forms the highest moral and professional standard which will serve to anchor us to the noblest conception of our calling, and help us not only to serve our patient faithfully, but shall command for us such a position as is accorded other branches of the healing art?

In the first place, it seems to me, that we must become a learned profession. By this statement I would not have you infer that we should necessarily cultivate the classics or even the sciences which may have no possible relation to our work; but we should have a fair knowledge of such learning as is necessary to make us intelligent above the ordinary. Particularly we should know the sciences which have any considerable bearing on the occupation we endeavor to follow; and, in addition, we should be able by a properly-disciplined mind to apply this knowledge in the solution of the many vexing problems which we encounter from day to day in the practice of our profession. To illustrate, we should be able to diagnose conditions of the mouth and teeth with reasonable accuracy and be able to outline the treatment and prognosticate a cure, from our knowledge of the sciences of Anatomy, Pathology, Pharmacology and Therapeutics involved. From our knowledge of the mechanical and physical laws involved, we should know how to construct a gold filling capable of withstanding the stress to which it may be subjected. Such knowledge enables us to so plan and construct anchorages and cavity forms that the frail walls of friable enamel shall be protected from mechanical, chemical and all other extraneous forces which might tend to destroy the usefulness of our best effort in filling building. These illustrations could be multiplied many times in all the wide range of opportunities involved in an up-to-date practice.

The realm of Physics, Chemistry and Biology are to be drawn on in almost every operation of any consequence which we undertake. Can any one then afford not to be familiar, at least, with the fundamentals of these sciences, and should he not be able to make application of their principles from his own knowledge, rather than, as is too often the case, use the principles in a sort of automatic way, because some one has said cavities should be so formed, or because experience may have taught us that such and such treatment will produce certain results? Is it not true that we do too large a part of our work empirically? Our teachers taught it so, or we found it worked well and we are satisfied to go on doing it so without stopping to reason out its philosophy, losing thereby one of the greatest benefits and pleasures in life,t hat of thinking for one's self. Learning for the sake of making a technical application of it is well worth our getting. And while we are getting wisdom and strength of character from learning, should we not also have in view the highest ideals of skill?

What is there that can so quickly kill all competition and professional strife as a burning desire in our profession to excel in skillful manipulation? Did any one ever hear of the best gold-filler, or most successful plate or crown and bridge-worker in the town, taking a notion that he would cut the prices lower than his co-laborers could afford, that he might do a larger business. Such a procedure might occur with an intensely-selfish person. The rule is that it is the man who is almost totally lacking in skill who resorts to unfair business methods to induce the unintelligent or selfish public to place its confidence in him. He is the man who thinks to use his mother wit to bring him success, and is too indolent to give the necessary time and effort to the cultivation of that skill which is the ideal of a true professional man.

It may be a question whether such a man ever entertained knowingly a worthy ideal or ambition. Such an one has no standards worthy of entertaining. A true mechanic is one who is never satisfied with a piece of work which does not only perform its function in a satisfactory way, but must be up to his highest conception of scientific construction and grace in outline. A man who has the skill to design and construct a beautiful watch, which will tell the time with scientific accuracy, is not only a genius, but if he can repeat such operations he becomes a professor.

What are the standards which should appeal to us more individually as *professional*? The first, and one that is most fundamental, is a consecration to the work, and a willingness to serve. We do not here mean a degraded or menial service, but a large and intelligent service which lifts the burdens of suffering from unfortunate mankind. Oftentimes such a service may be trifling, so far as the learning and skill required are concerned; and it may also require little time or physical expense, but yet be so grateful to the afflicted that angels could do no more. These services are not unusally measured in quantity, but in quality. The kind words and gentle touch of a dignified and decorous dentist, whose cleanly habits and cheerful manners put the impassive stoic as well as the most sensitive child at once *en rapport*, are more worthy ideals than the unfeeling and brusque business methods which too often characterize the attitude of our profession. There is probably no other profession where so much pain is inflicted, and too often with no intelligent or well-directed effort to shield the patient. When our attention is called to the fact that we are hurting our patient dreadfully, we are too much inclined to make some trifling rejoinder and proceed to do even worse than before. We lose acuteness of sympathy from frequent repetition of similar experiences, and unless we have something to prevent it, we are certain to become hardened to appeals for sympathy, and our service loses its professional character and becomes menial and selfish. We would not be understood here as advocating insufficient proceedings because painful, as this will most certainly degenerate to trifling expedients and inadequate service; but when heroic and painful services are required, they should be administered in the most humane and sympathetic manner practicable. The selfish or incompetent dentist will not be willing to make the sacrifice of time necessary to accomplish this, if indeed, he be possessed of the necessary intelligence. It is an ideal that should go hand in hand with another element of professional service that is characteristic, and that is the establishment of confidential relations with our patients. That dentist who by the breadth of his learning and wealth of his attainments hopes to draw people to him regardless of his morals and sympathetic nature is doomed sooner or later to business failure and disappointment. Personal qualitites of an acceptable character are absolutely essential to professional success. A man should be temperate and able to hold himself in absolute control under the most trying circumstances.

It is unecessary to say that sobriety, purity of life, and other of the common virtues have a large place in establishing the most lasting friendships contributing materially to professional success. All forms of quackery are not only dishonorable, and of course to be religiously avoided.

If indulged at all, they become demoralizing, retroacting influences, demeaning to the whole life. Such influences will, of course, preclude the establishment of those enduring professional relations which are necessary to make one feel that his life's work is akin to that which is divine and therefore satisfactory to the soul. In a word, the true professional standard is one of highest morals and can never be attained without the complete surrender of all impure and unworthy motives, and the entire consecration of one's whole life to its attainment. No one should esteem his calling merely for what it brings of this world's comfort to him, but because it really appeals to his best self and brings full compensation for all the sacrifices it calls upon him to make. Can there be any doubt of the success in a pecuniary sense of one who strives conscientiously to realize such an ideal?

When we come to consider the commercial aspect of our subject, we find it somewhat difficult to idealize. At present there is no definite basis of compensating professional services. The old fee system, which has come down to us from the ages, has some features that commend it. As originally practiced, it was based on the gratitude of the patient and his ability to pay. Owing to the fact that in these days patients are not sufficiently appreciative of the spirit which in former times made this system possible it has fallen largely into disuse, and we have adopted the more commercial system of pay for what you get. The only remnant of it now known is to be found in the agreement of dentists in certain localities to a uniform fee bill, which they try to enforce. A charge of fifty cents, for instance, is the almost universal fee for extracting a tooth. It is so well established that any variation from it will subject the dentist to the suspicion of some kind of unfair dealing by his patients. But outside of this and some forms of plate-work each man feels at perfect liberty to set his fees at whatever rate he desires. One man according to the value which he places on his own services; another on his necessities; and still others for various reasons at various standards, while some it is believed base them on their opportunity or ability of the patient to pay.

There is nothing unprofessional in any one setting his fees at such a standard as may please himself so long as they are no lower than those which are customary in the place where he locates. If a lower scale is adopted it works injury to one's associates and leads to professional demoralization. It is a matter of much doubt whether a professional man has a moral right to make special fees for perticular patients to meet his own financial exigencies. There can of course be no fault found with exacting like fees for similar work for all persons. A physician recently told me that a friend approached him with an inquiry as to whether he was not treating lawyer Blank's daughter; when informed that he was, he was asked if he would not be as considerate as he could as to his fee for the friend said he would probably have to pay it, as lawyer Blank was trying a case for him. It is not fair nor professional to tax a patient two or three times the regular fee for any service, simply because he happens to be rich or free with his money. Of course when a patient has money and needs a service requiring skillful service, an adequate fee should be exacted; to charge a patient an exorbitant fee simply because he is rich is not honorable or professional, but is essentially degrading personally and to the profession. There can be but one proper basis for adjusting professional fees and that one should recognize the rights of both the patient and the professional man. Custom usually makes a fee basis, but this is not always just, for one man will accomplish a service in a superior manner while another simply gets it off his hands. The values are wholly unequal and should never be on the same basis. It is difficult to demonstrate such facts to patients, hence the injustice often falls upon the man who has done a good service rather than on the one who has simply coverd his faults. An intelligent public will generally discriminate and eventually do justice to the man who does the best he knows how, and who makes it clear that he uses his time and means to prepare himself to serve his people in the most advantageous manner.

Dentists seldom get rich, and what is worse they often are not good business men. A professional man ought not to expect to get rich, for if he devotes his mind to the many vexatious problems which come to him daily, he will not have much time for devising ways of accumulating property.

Every dentist should carefully estimate the value of his services and have some fixed business standards so that his patients may know what to expect from him in the way of bills for services. This should not mean that no variations are permissible, as occasions will frequently arise that call for material modifications. It should be a principle when these things are necessary that the patient should be advised in advance of the facts.

In all financial transactions a standard of honesty and consideration for both patient and dentist should prevail; and it is equally important that the dentist feels compensated with an adequate fee, as that the patient should feel that he has secured a good service for a reasonable compensation. Any system which can be devised that will accomplish this object ought to be equitable and therefore eminently professional.

DISCUSSION.

Dr. P. F. Hines: There is no question but we are considerably demoralized on the question of fees for services rendered our patients. The results of competition and ignorance of the people have much to do with it, but dentists do not handle this matter in a business way, they allow themselves to be influenced by their patients or competitors to their own harm. The first and important factor should be that of the quality of the service, and when it is of high character, or require extraordinary skill, it should be correspondingly compensated. There are some patients who feel that a large fee always assures better service and are suspicious of inferior work when the fee is small. Again other people object to a compensating fee because they have no appreciation of values. With such persons explanations should always be made that they may not be dissatisfied.

In some kinds of work, like extracting a tooth or making a plate or crown, the same fee must be charged in every case or the patient will think the dentists is chargnig him more than he did his neighbor who paid a smaller fee for a similar service. In other departments patients can not make comparisons so easily. And yet it is worth much more to make plates for some people than it is for others, and patients should be enlightened on this subject, or at least given an opportunity to select the better forms of platework regardless of fees. I do not mean to say that work in quality should be graded on the basis of the fee paid. All work should be of the best grade one is capable of making, and where there is a requirement for extra work or material and skill, the patient should be made to appreciate the difference. There are some people who will endeavor to get all the service they can for the least possible fee, and sometimes attribute failures of work to the dentist which belong to themselves. No standard of dealing can be made for such cases, each one must be met on its merits. A patient drops his plate or steps on it and breaks it, comes with the story that he broke it eating soft bread; he should be very diplomatically handled to avoid alienating his favors, and at the same time he should not be given a chance to depreciate your own rights, to say nothing of encouraging in him an evil tendency which may make a pauper or a rascal of him. If the dentists of a neighborhood were more friendly it would be much easier to establish uniform standards of fees at least. But uniformity in standards of attainment can not be expected.

Dr. Warboys: If we are honest with ourselves we shall be also with our patients. We sometimes promise our patients more than we are able to perform, and as a consequence bring disappointment to our patients and dissatisfaction upon ourselves. We think we are smart enough to know what is best for our patients and base our judgment oftentimes upon our estimate of the size of the fee fhe patient is willing to pay. We tell a patient for instance that a rubber plate is the best thing for them, when we know it is not. The conditions of the mouth for instance clearly call for some form of a metal plate, but we don't want to make it, or perhaps the patient may say that it is too expensive. We should enlighten every patient as to values of the kinds of work, and give them the opportunity of making their selection after being fully informed.

We should always be careful to make good any faulty workmanship. If it is detected before delivery it should be remedied at once, or if it is returned by the patient it should be cheerfully made good. A good way to keep on good terms with our confreres is to be careful to uphold them rather than to discredit them when their patients seek you for advise or service, You will frequently have opportunities to make over plates made by another dentist, and you will naturally try to avoid making the same mistakes he has mads and perhaps you will succeed where he has failed. This may be because you take unusual pains to do your work the best you know how. It should not, however, be an occasion for you to discredit the effort of your associate as he did not have the same information to work from that you had.

The standard for prosthetic dentistry in most places is miserably low. A vulcanite plate can not be made as it should be for ten dollars, and yet that is the average price for rubber plates in most places. People expect the dentist to make a rubber plate that will be as usefull as the natural teeth were, and will look better than the natural teeth ever did, and all for ten dollars, it matters not what difficulties may have to be overcome in making an acceptable plate. There is a great difference in mouths, and it is worth more money to make a plate that will fit some mouths than it is to make the same kind of a plate for another mouth, which lends itself readily to ordinary methods. This is where skill and experience count, but patients don't discriminate on this basis.

Of course a poor man is entitled to as good service as a rich one, and some discrimination should be made in his favor. The poor man should have all and more than he pays for; and this does not imply that a rich man should not also have all that he pays for. If he pays more than the usual fee he should have more than usual attention and service. There is a class of patients which try our patience; they are the dead beats and people who do not think it is any expense or trouble for you to wait on them. They will ask you to get up out of bed to serve them and go away and forget to pay you for your trouble. I am perfectly willing to inconvenience myself to relieve any one from sufferng, but I feel better when some acknowledgement is made for the service.

Dr. Hodges: I make it a point never to turn away a patient who is in distress that I can relieve. I sometimes get caught by some one who forgets to recompense me. But I keep a dead-beat list and follow each one up as occasion offers, and always refuse to do further work for such a person unless he liquidates his account. If he should be so situated as to make this impracticable, I am always willing to do what is necessary to keep his teeth comfortable. If we allow patients to cheat us, they don't think any more of us, and it has a tendency to discredit us. I am willing to render benevolent service when it is necessary, but not to be imposed upon by rascals.

